# Magnetic field effects on plant growth, development, and evolution

**DOI:** 10.3389/fpls.2014.00445

**Published:** 2014-09-04

**Authors:** Massimo E. Maffei

**Affiliations:** Department of Life Sciences and Systems Biology, Innovation Centre, University of TurinTurin, Italy

**Keywords:** geomagnetic field, plant responses, evolution, magnetoreception, cryptochrome

## Abstract

The geomagnetic field (GMF) is a natural component of our environment. Plants, which are known to sense different wavelengths of light, respond to gravity, react to touch and electrical signaling, cannot escape the effect of GMF. While phototropism, gravitropism, and tigmotropism have been thoroughly studied, the impact of GMF on plant growth and development is not well-understood. This review describes the effects of altering magnetic field (MF) conditions on plants by considering plant responses to MF values either lower or higher than those of the GMF. The possible role of GMF on plant evolution and the nature of the magnetoreceptor is also discussed.

## Introduction

A magnetic field (MF) is an inescapable environmental factor for plants on the Earth. During the evolution process, all living organisms experienced the action of the Earth's MF (geomagnetic, GMF), which is a natural component of their environment. GMF is steadily acting on living systems, and is known to influence many biological processes. There are significant local differences in the strength and direction of the earth's magnetic (geomagnetic) field. At the surface of the earth, the vertical component is maximal at the magnetic pole, amounting to about 67 μT and is zero at the magnetic equator. The horizontal component is maximal at the magnetic equator, about 33 μT, and is zero at the magnetic poles (Kobayashi et al., 2004). A MF is usually measured in terms of its magnetic induction **B** whose unit is given in Tesla (T), defined as:

$$T=\frac{V•s}{m^{2}}=\frac{Wb}{m^{2}}=\frac{N•s}{c•m}$$

The dimension of the “magnetic flux” (φ) in SI units is Weber (Wb) (φ = *Tm*^2^ = *Wb*).

The MF strength at the Earth's surface ranges from less than 30 μT in an area including most of South America and South Africa (the so called south Atlantic anomaly) to almost 70 μT around the magnetic poles in northern Canada and south of Australia, and in part of Siberia (Occhipinti et al., 2014). Most of the MF observed at the Earth's surface has an internal origin. It is mainly produced by the dynamo action of turbulent flows in the fluid metallic outer core of the planet, while little is due to external MFs located in the ionosphere and the magnetosphere (Qamili et al., 2013). It is the presence of the GMF that, through the magnetosphere, protects the Earth, together with its biosphere, from the solar wind deflecting most of its charged particles (Occhipinti et al., 2014).

The literature describing the effects of weak MFs on living systems contains a plethora of contradictory reports, few successful independent replication studies and a dearth of plausible biophysical interaction mechanisms. Most such investigations have been unsystematic, devoid of testable theoretical predictions and, ultimately, unconvincing (Harris et al., 2009). The progress and status of research on the effect of MF on plant life have been reviewed in the past years (Phirke et al., 1996; Abe et al., 1997; Volpe, 2003; Belyavskaya, 2004; Bittl and Weber, 2005; Galland and Pazur, 2005; Minorsky, 2007; Vanderstraeten and Burda, 2012; Occhipinti et al., 2014).

Krylov and Tarakonova (1960) were among the first to report on MF effects on plants. They proposed an auxin-like effect of the MF on germinating seeds, by calling this effect magnetotropism. The auxin-like effect of MF was also suggested to explain ripening of tomato fruits (Boe and Salunkhe, 1963). There was evidence that the root-growth response was not directly heliotropic but rather magnetotropic or geo-magnetotropic. Observation of the roots of a number of other plants suggested that some inherent factor within a species or even within a variety of a species could also be necessary before the tropism became manifest (Pittman, 1962). Because of the insufficient understanding of the biological action of MFs and its mechanism, it is rare to document the magnetic environment as a controlled factor for scientific experiment. Anyway, from these pioneering studies on, we can consider two experimental approaches aimed to evaluate the physiological responses of plant exposed to either weak or strong MFs.

## Exposure of plants to a MF intensity lower than the geomagnetic field

The term weak or low MF is generally referred to the intensities from 100 nT to 0.5 mT, whereas superweak or conditionally zero (the so called magnetic vacuum) is related to MFs below 100 nT. Investigations of low MF effects on biological systems have attracted attention of biologists for several reasons. For instance, interplanetary navigation will introduce man, animals and plants in magnetic environments where the MF is near 1 nT. It is known that a galactic MF induction does not exceed 0.1 nT, in the vicinity of the Sun (0.21 nT), and on the Venus surface (3 nT) (Belov and Bochkarev, 1983). This brought a new wave of interest in MF role in regulating plant growth and development (Belyavskaya, 2004). In laboratory, low MF have been created by different methods, including shielding (surrounding the experimental zone by ferromagnetic metal plates with high magnetic permeability, which deviate MF and concentrate it in the metal) and compensating (by using Helmholtz coils). In general, developmental studies on plant responses have been performed at various MF intensities. Early in 1963, it was found that a MF of relatively low intensity could be effective in stimulating or initiating plant growth responses (Pittman, 1963). Since then, a few experiments evaluated the effects of reduced MF conditions if compared to those performed by using high intensity MFs.

### Effects on plant development

Sunflower (*Helianthus annuus*) seedlings exposed to 20 μT vertical MF showed small, but significant increases in total fresh weights, shoot fresh weights, and root fresh weights, whereas dry weights and germination rates remained unaffected (Fischer et al., 2004).

Pea (*Pisum sativum*) epicotyls were longer in low MF (11.2 ± 4.2 mm, *n* = 14) when compared to normal geomagnetic conditions (8.8 ± 4.0 mm, *n* = 12) (Yamashita et al., 2004). Elongation of pea epicotyl was confirmed, by microscopic observation of sectioned specimen, to result from the elongation of cells and osmotic pressure of seedlings was significantly higher in low MF than controls. This observation suggests that the promotion of cell elongation under low MF may relate to an increase of osmotic pressure in the cells (Negishi et al., 1999). Furthermore, pea seedlings showed ultrastructural peculiarities such as a noticeable accumulation of lipid bodies, development of a lytic compartment (vacuoles, cytosegresomes, and paramural bodies), and reduction of phytoferritin in plastids. Mitochondria were the most sensitive organelle to low MF treatment and their size and relative volume in cells increased, matrix was electron-transparent, and cristae reduced. It was also observed that low MF effects on ultrastructure of root cells were due to disruptions in different metabolic systems including effects on Ca^2+^ homeostasis (Belyavskaya, 2001).

In broad bean (*Vicia faba*) seedlings, low MF intensities of 10 and 100 μT at 50 or 60 Hz were observed to alter membrane transport processes in root tips (Stange et al., 2002), whereas seeds of soybean (*Glycine max*) exposed to pulsed MF of 1500 nT at 0.1, 1.0, 10.0, and 100.0 Hz for 5 h per day for 20 days, induced by enclosure coil systems, significantly increased the rate of seed germination, while 10 and 100 Hz pulsed MFs showed the most effective response (Radhakrishnan and Kumari, 2013). Treatment with MF also improved germination-related parameters like water uptake, speed of germination, seedling length, fresh weight, dry weight, and vigor indices of soybean seeds under laboratory conditions (Shine et al., 2011).

Controversial data have also been reported. The exposure to near null MF of different *in vitro* cultures of various species of the genus *Solanum* was either stimulating or inhibiting the growth of *in vitro* plants. The effect was apparently also dependent on the species, genotype, type of initial explant, treatment duration, or even culture medium (Rakosy-Tican et al., 2005).

By using ferromagnetic shields, the influence of weak, alternating MF, which was adjusted to the cyclotron frequency of Ca^2+^ and K^+^ ions, was studied on the fusion of tobacco (*Nicotiana tabacum*) and soybean protoplasts. It was observed that in these conditions protoplasts fusion increased its frequency 2–3 times with the participation of calcium ions in the induction of protoplast fusion (Nedukha et al., 2007). The observations of the increase in the [Ca^2+^]_cyt_ level after exposure to very low MF suggests that Ca^2+^ entry into the cytosol might constitute an early MF sensing mechanism (Belyavskaya, 2001).

When wheat (*Triticum aestivum*) seeds were treated with low-frequency magnetic MF at the stage of esterase activation during seed swelling, the activation of esterases was enhanced by changing qualitatively the time course of the release of reaction products into the medium. These results helped to explain unusual dependences of biological effects on the amplitude of the electromagnetic field (EMF), including the atypical enhancement of these effects by the action of weak low-frequency fields (Aksenov et al., 2000). A two-layer permalloy magnetic screen was used to test the effects of a wide range of low MF (from 20 nT to 0.1 mT) on 3–5 day old wheat seedlings. It was observed that seedlings grew slower than controls (Bogatina et al., 1978).

Barley (*Hordeum vulgare*) seedlings grown in Helmholtz coils with a 10 nT MF intensity showed a decrease in fresh weight of shoots (by 12%) and roots (by 35%), as well as dry weight of shoots (by 19%) and roots (by 48%) in comparison with GMF controls. From this pioneer study it was concluded that very low MF was capable of delaying both organ formation and development (Lebedev et al., 1977).

The effect of a combined MF at the resonance frequency of Ca^2+^ ions inside a μ-metal shield and the altered gravitropic reaction of cress (*Lepidium sativum*) roots was performed to evaluate the structure and functional organization of root cap statocytes. The experimented conditions were observed to change normally positively gravitropic cress root to exhibit negative gravitropism (Kordyum et al., 2005).

Artificial shielding of GMF caused a significant decrease in the cell number with enhanced DNA content in root and shoot of onion (*Allium cepa*) meristems. Furthermore, the uncytokinetic mitosis with formation of binuclear and then tetranuclear cells as well as a fusion of normal nuclei resulting in appearance of giant cells with vast nuclei seems to dominate in very low MF conditions (Nanushyan and Murashov, 2001).

Changes in the ultrastructural organization of some organelles and cellular compartments, alterations in condensed chromatin distribution and reduction in volume of granular nucleolus component with the appearance of nucleolus vacuoles were also found in several other species exposed to very low MF, indicating a decrease in activities of rRNA synthesis in some nucleoli (Belyavskaya, 2004 and references cited therein).

### Effects on transition to flowering

Near-null MF can be produced by three mutually perpendicular couples of Helmholtz coils and three sources of high-precision direct current power, which can counteract the vertical, north–south and east–west direction components of the geomagnetic field (GMF) (Xu et al., 2012).

Although the functions of cryptochrome have been well-demonstrated for *Arabidopsis thaliana*, the effect of the GMF on the growth of Arabidopsis and its mechanism of action are poorly understood. In Arabidopsis seedlings grown in a near-null MF flowering was delayed by ca. 5 days compared with those grown in the GNF. Moreover, PCR analyses of three cryptochrome-signaling-related genes, *PHYB*, *CO*, and *FT* also changed; the transcript level of *PHYB* was elevated ca. 40%, and that of *CO* and *FT* was reduced ca. 40 and 50%, respectively. These data suggest that the effects of a near-null MF on Arabidopsis might be cryptochrome-related, which may be revealed by a modification of the active state of cryptochrome and the subsequent signaling cascade (Xu et al., 2012). Moreover, the biomass accumulation of plants in the near-null MF was significantly suppressed at the time when plants were switching from vegetative growth to reproductive growth compared with that of plants grown in the local GMF, which was caused by the delay in the flowering of plants in the near-null MF. These resulted in a significant reduction of about 20% in the harvest index of plants in the near-null MF compared with that of the controls. Therefore, the removal of the local GMF negatively affects the reproductive growth of Arabidopsis, which thus affects the yield and harvest index (Xu et al., 2013).

## Exposure of plants to MF intensities higher than the geomagnetic filed

A consistent number of papers described the effect of MF intensities higher than the GMF levels. In general, intensities higher than GMF relate to values higher than 100 μT. As summarized in Table 1, experimental values can reach very high MF levels, ranging from 500 μT up to 15 T. Most of the attention has been focused on seed germination of important crops like wheat, rice and legumes. However, many other physiological effects on plants of high MF described plant responses in terms of growth, development, photosynthesis, and redox status.

**Table 1:** **Summary of magnetic field (MF) effects on plants**.

**Plant species**	**Plant organ**	**Effect**	**MF intensity**	**References**
**EXPOSURE TO MF VALUES LOWER THAN THOSE OF THE GMF**				
*Actinidia deliciosa*	Pollen	Release of internal Ca^2+^	10 μT	Betti et al., 2011
*Allium cepa*	Root and shoot	Decrease in the cell number with enhanced DNA content	<GMF	Nanushyan and Murashov, 2001; Belyavskaya, 2004 and references cited therein
*Arabidopsis thaliana*		Delayed flowering Reproductive growth	Near null	Xu et al., 2012, 2013
*Glycine max*	Protoplasts Seeds	Increased protoplasts fusion	<GMF	Nedukha et al., 2007
		Seed germination	1500 nT	Radhakrishnan and Kumari, 2013
*Helianthus annuus*	Seedlings	Increases in fresh weight	20 μT	Fischer et al., 2004
*Hordeum volgare*	Seedlings	Decrease in fresh weight	10 nT	Lebedev et al., 1977
*Lepidium sativum*	Roots	Negative gravitropism	<GMF	Kordyum et al., 2005
*Nicotiana tabacum*	Protoplasts	Increased protoplasts fusion	<GMF	Nedukha et al., 2007
*Pisum sativum*	Epicotyl	Promotion of cell elongation; ultrastructural peculiarities increase in the [Ca^2+^]_cyt_ level	<GMF	Negishi et al., 1999; Belyavskaya, 2001; Yamashita et al., 2004
*Solanum* spp.	*In vitro* cultures	Stimulation/inhibition of growth	<GMF	Rakosy-Tican et al., 2005
*Triticum aestivum*	Seeds and seedlings	Activation of esterases reduction of growth	from 20 nT to 0.1 mT	Bogatina et al., 1978; Aksenov et al., 2000
*Vicia faba*	Root tips	Alter membrane transport processes	10 and 100 μT	Stange et al., 2002
**EXPOSURE TO MF VALUES HIGHER THAN THOSE OF THE GMF**				
*Abelmoschus esculentus*	Seed	Promotion of germination	99 mT	Naz et al., 2012
*Allium ascalonicum*	Seedlings	Increased lipid peroxidation and H_2_O_2_ levels	7 mT	Cakmak et al., 2012
*Arabidopsis thaliana*	Seedlings	Enhanced blue light-dependent phosphorylations of CRY1 and CRY2; hypocotyl growth	500 μT	Harris et al., 2009; Xu et al., 2014
	Callus culture		15 T	Weise et al., 2000
		Amyloplast displacement		Manzano et al., 2013
		Diamagnetic levitation		Herranz et al., 2013
		Proteomic alterations		Paul et al., 2006
		Induced expression of the Adh/GUS transgene in the roots and leaves		
*Beta vulgaris*	Seedlings	Increased root and leaf yield	5 mT	Rochalska, 2008 Rochalska, 2005
		Increased chlorophyll content		Rochalska, 2005
*Carica papaya*	Pollen	Increased pollen germination	>GMF	Alexander and Ganeshan, 1990
*Catharanthus roseus*	Protoplast	Effect on cell wall	302 mT	Haneda et al., 2006
*Cicer arietinum*	Seed	Promotion of germination	0–250 mT	Vashisth and Nagarajan, 2008
	Root	Increase in root length, surface area and volume		
*Coffea arabica*	Seedlings	Decrease of SOD, CAT, and APX activities	2 mT	Aleman et al., 2014
*Cryptotaenia japonica*	Seed	Promotion of germination	500, 750 μT	Kobayashi et al., 2004
*Cucumis sativus*	Seedlings	Increase in superoxide radicals and H_2_O_2_	100–250 mT	Bhardwaj et al., 2012
*Desmodium gyrans*	Leaf	Reduced rhythmic leaflet movements	50 mT	Sharma et al., 2000
*Dioscorea opposita*	Seedling	Increased root length and number	2× GMF	Li, 2000
*Fragaria vesca*	Plantlets	Increased fruit yield per plant	0.096, 0.192 and 0.384 T	Esitken and Turan, 2004
*Glycine max*	Seedlings	Reduction of O_2_-radical level	150,200 mT	Baby et al., 2011; Radhakrishnan and Kumari, 2012, 2013; Shine et al., 2012
		Reactive oxygen species production		
		Increased Rubisco		Shine et al., 2011
*Helianthus annuus*	Seedlings	Increased seedling dry weight, root length, root surface area and root volume	50, 200 mT	Vashisth and Nagarajan, 2010
		Increased activities of α-amylase, dehydrogenase and protease		
*Helianthus annuus*	Seedlings	Increased chlorophyll concentration	>GMF	Turker et al., 2007
*Helix aspesa*	Seedlings	Oxidative burst	50-Hz	Regoli et al., 2005
*Hordeum vulgare*	Seedlings	Increases in length and weight	125 mT	Martinez et al., 2000
*Leymus chinensis*	Seedlings	Increased peroxidase activity	200,300 mT	Xia and Guo, 2000
*Oryza sativa*	Seed	Reduction of germination	125,250 mT	Florez et al., 2004
*Paulownia fortunei*	Tissue cultures	Increased regeneration capability	2.9–4.8 mT	Yaycili and Alikamanoglu, 2005
*Paulownia tomentosa*	Tissue cultures	Increased regeneration capability	2.9–4.8 mT	Yaycili and Alikamanoglu, 2005
*Petroselinum crispum*	Cells	Effects on CAT and APX activity	30 mT	Rajabbeigi et al., 2013
*Phaseolus vulgaris*	Seeds	Promotion of germination	2 or 7 mT	Sakhnini, 2007; Cakmak et al., 2010
		Increased chlorophyll emission fluorescence	3 100,160 mT	Jovanic and Sarvan, 2004
*Pisum sativum*	Seed	Promotion of germination	60,120,180 mT	Iqbal et al., 2012
	Seedlings	Increased length and weight	125, 250 mT	Carbonell et al., 2011
		Induction of SOD activity		Polovinkina et al., 2011
*Raphanus sativus*	Seedlings	Suppression of SOD and CAT activities	185–650 μT	Serdyukov and Novitskii, 2013
		Reduced CO_2_ uptake	500 μT	Yano et al., 2004
		Stimulation of lipid synthesis		Novitskaya et al., 2010; Novitskii et al., 2014
*Solanum lycopersicum*	Seed	Promotion of germination	160–200 mT	De Souza et al., 2010; Poinapen et al., 2013a
*Solanum lycopersicum*	Seed	Promotion of germination	160–200 mT	De Souza et al., 2010; Poinapen et al., 2013a
	Shoots	Effect on gravitropismo		
		Magnetophoretic curvature		Hasenstein and Kuznetsov, 1999
		Increased mean fruit weight, yield per plant and per area		De Souza et al., 2006
		Geminivirus and early blight and a reduced infection rate		
*Solanum tuberosum*	Seedlings	Amyloplast displacement	4 mT	Hasenstein et al., 2013
	Plantlets	Growth promotion and enhancement of CO_2_ uptake enhanced lipid order		Iimoto et al., 1998
				Poinapen et al., 2013b
*Taxus chinensis*	Suspension culture	Promotion of taxol production	3.5 mT	Shang et al., 2004
*Tradescantia* spp.	Inflorescence	Pink mutations in stamen hair cells	0.16, 0.76, 0.78 T	Baum and Nauman, 1984
*Triticum aestivum*	Seed	Promotion of germination	4 or 7 mT; 30-mT	Cakmak et al., 2010
	Seedlings	Amyloplast displacement increased catalase but reduced peroxidase activity	30-mT	Hasenstein et al., 2013
				Payez et al., 2013
*Vicia faba*	Plantlets	Accumulation of ROS	15 mT	Jouni et al., 2012
		Modification of catalase and MAPK; accumulation of H_2_O_2_	30 mT	Haghighat et al., 2014
*Vigna radiata*	Seed	Promotion of germination	87 to 226 mT	Mahajan and Pandey, 2014
	Seedlings	Decrease of malondialdehyde, H_2_O_2_ and O^−^_2_, and increase of NO and NOS activity	600 mT	Chen et al., 2011
*Zea mays*	Seed	Promotion of germination		Bilalis et al., 2012
	Seedlings	Increase of fresh weight	125,250 mT	Florez et al., 2007
		Amyloplast displacement		Hasenstein et al., 2013
		Decreased levels of hydrogen peroxide and antioxidant defense system enzymes	100,200 mT	Anand et al., 2012
				Shine and Guruprasad, 2012
		Reduction of antioxidant enzymes		Turker et al., 2007; Javed et al., 2011; Anand et al., 2012
		Increased stomatal conductance and chlorophyll content	100,200 mT	
			100,200 mT	

### Effects on germination

A MF applied to dormant seeds was found to increase the rate of subsequent seedling growth of barley, corn (*Zea mays*), beans, wheat, certain tree fruits, and other tree species. Moreover, a low frequency MF (16 Hz) can be used as a method of post-harvest seed improvement for different plant species, especially for seeds of temperature sensitive species germinating at low temperatures (Rochalska and Orzeszko-Rywka, 2005).

Seeds of hornwort (*Cryptotaenia japonica*) exposed to sinusoidally time-varying extremely low frequency (ELF) MFs (AC fields) in combination with the local GMF showed a promoted activity of cells and enzymes in germination stage of the seed. This suggests that an optimum ELF MF might exist for the germination of hornwort seeds under the local GMF (Kobayashi et al., 2004). The application of AC field also promoted the germination of bean (*Phaseolus vulgaris*) seeds (Sakhnini, 2007).

In seeds of mung bean (*Vigna radiata*), exposed in batches to static MFs of 87 to 226 mT intensity for 100 min, a linear increase in germination magnetic constant with increasing intensity of MF was found. Calculated values of mean germination time, mean germination rate, germination rate coefficient, germination magnetic constant, transition time, water uptake, indicate that the impact of applied static MF improves the germination of mung beans seeds even in off-season (Mahajan and Pandey, 2014).

The seeds of pea exposed to full-wave rectified sumusoidal non-uniform MF of strength 60, 120, and 180 mT for 5, 10, and 15 min prior to sowing showed significant increase in germination. The emergence index, final emergence index and vigor index increased by 86, 13, and 205%, respectively. Furthermore, it was found that exposure of 5 min for MF strengths of 60 and 180 mT significantly enhanced the germination parameters of the pea and these treatments could be used practically to accelerate the germination in pea (Iqbal et al., 2012).

MF application with a strength from 0 to 250 mT in steps of 50 mT for 1–4 h significantly enhanced speed of germination, seedling length and seedling dry weight compared to unexposed control in chickpea (*Cicer arietinum*). It was also found that magnetically treated chickpea seeds may perform better under rainfed (un-irrigated) conditions where there was a restrictive soil moisture regime (Vashisth and Nagarajan, 2008).

Different intensities of static MF (4 or 7 mT) were tested on seed germination and seedling growth of bean or wheat seeds in different media having 0, 2, 6, and 10 atmosphere (atm) osmotic pressure prepared with sucrose or salt. The application of both MFs promoted the germination ratios, regardless of increasing osmotic pressure of sucrose or salt. The greatest germination and growth rates in both plants were from the test groups exposed to 7 mT (Cakmak et al., 2010).

Seeds of wheat were imbibed in water overnight and then treated with or without a 30 mT static magnetic field (SMF) and a 10 kHz EMF for 4 days, each 5 h. Exposure to both MF increased the speed of germination, compared to the control group, suggesting promotional effects of EMFs on membrane integrity and growth characteristics of wheat seedlings (Payez et al., 2013).

Pre-sowing treatment of corn seeds with pulsed EMFs for 0, 15, 30, and 45 min improved germination percentage, vigor, chlorophyll content, leaf area, plant fresh and dry weight, and finally yields. Seeds that have been exposed to MF for 30 and 45 min have been found to perform the best results with economic impact on producer's income in a context of a modern, organic, and sustainable agriculture (Bilalis et al., 2012).

Various combinations of MF strength and exposure time significantly improved tomato (*Solanum lycopersicum*) cv. Lignon seed performance in terms of reduction of time required for the first seeds to complete germination, time to reach 50% germination, time between 10 and 90% germination with increasing germination rate, and increased germination percentage at 4 and 7 days, seedling shoot and root length compared to the untreated control seeds. The combinations of 160 mT for 1 min and 200 mT for 1 min gave the best results (De Souza et al., 2010). Higher germination (about 11%) was observed in magnetically-exposed tomato var. MST/32 seed than in non-exposed ones, suggesting a significant effect of non-uniform MFs on seed performance with respect to RH (Poinapen et al., 2013a).

The effect of pre-sowing magnetic treatments was investigated on germination, growth, and yield of okra (*Abelmoschus esculentus* cv. Sapz paid) with an average MF exposure of 99 mT for 3 and 11 min. A significant increase (*P* < 0.05) was observed in germination percentage, number of flowers per plant, leaf area, plant height at maturity, number of fruits per plant, pod mass per plant, and number of seeds per plant. The 99 mT for 11 min exposure showed better results as compared to control (Naz et al., 2012).

However, contrasting results have also been reported. For instance, the mean germination time of rice (*Oryza sativa*) seeds exposed to one of two MF strengths (125 or 250 mT) for different times (1 min, 10 min, 20 min, 1 h, 24 h, or chronic exposure) was significantly reduced compared to controls, indicating that this type of magnetic treatment clearly affects germination and the first stages of growth of rice plants (Florez et al., 2004).

### Effects on cryptochrome

The blue light receptor cryptochrome can form radical pairs after exposure to blue light and has been suggested to be a potential magnetoreceptor based on the proposition that radical pairs are involved in magnetoreception. Nevertheless, the effects of MF on the function of cryptochrome are poorly understood. When Arabidopsis seedlings were grown in a 500 μT MF and a near-null MF it was found that the 500 μT MF enhanced the blue light-dependent phosphorylations of CRY1 and CRY2, whereas the near-null MF weakened the blue light-dependent phosphorylation of CRY2 but not CRY1. Dephosphorylations of CRY1 and CRY2 in the darkness were slowed down in the 500 μT MF, whereas dephosphorylations of CRY1 and CRY2 were accelerated in the near-null MF. These results suggest that MF with strength higher or weaker than the local GMF affects the activated states of cryptochromes, which thus modifies the functions of cryptochromes (Xu et al., 2014). Moreover, the magnitude of the hyperfine coupling constants (A^(iso)^_max_ = 17.5 G) suggests that artificial MFs (1–5 G) involved in experiments with Arabidopsis can affect the signal transduction rate. On the other hand, hyperfine interactions in the FADH^▪^-Trp^▪+^ biradicals are much stronger than the Zeeman interaction with the MF of the Earth (≈0.5 G). Therefore, an alternative mechanism for the bird avian compass has been proposed very recently. This mechanism involves radicals with weaker hyperfine interactions (O^▪−^_2_ and FADH^▪^), and thus, it could be more plausible for explaining incredible sensitivity of some living species to even tiny changes in the MF (Izmaylov et al., 2009).

However, contrasting results were obtained when the intensity of the ambient MF was varied from 33–44 to 500 μT. According to Ahmad et al. (2007) there was an enhanced growth inhibition in Arabidopsis under blue light, when cryptochromes are the mediating photoreceptor, but not under red light when the mediating receptors are phytochromes, or in total darkness. Hypocotyl growth of Arabidopsis mutants lacking cryptochromes was unaffected by the increase in magnetic intensity. Additional cryptochrome-dependent responses, such as blue-light-dependent anthocyanin accumulation and blue-light-dependent degradation of CRY2 protein, were also enhanced at the higher magnetic intensity. On the contrary, Harris et al. (2009) by using the experimental conditions chosen to match those of the Ahmad study, found that in no case consistent, statistically significant MF responses were detected. For a more comprehensive discussion on cryptochromes see below.

### Effects on roots and shoots

Increased growth rates have been observed in different species when seeds where treated with increased MF. Treated corn plants grew higher and heavier than control, corresponding with increase of the total fresh weight. The greatest increases were obtained for plants continuously exposed to 125 or 250 mT (Florez et al., 2007). A stimulating effect on the first stages of growth of barley seeds was found for all exposure times studied. When germinating barley seeds were subjected to a MF of 125 mT for different times (1, 10, 20, and 60 min, 24 h, and chronic exposure), increases in length and weight were observed (Martinez et al., 2000). Pants of pea exposed to 125 or 250 mT stationary MF generated by magnets under laboratory conditions for 1, 10, and 20 min, 1 and 24 h and continuous exposure were longer and heavier than the corresponding controls at each time of evaluation. The major increases occurred when seeds were continuously exposed to the MF (Carbonell et al., 2011).

By treating with twice gradient MF *Dioscorea opposita* it was found that they could grow best in the seedling stage. Compared with the control, the rate of emergence increased by 39%, root number increased by 8%, and the average root length increased by 2.62 cm (Li, 2000). The 16 Hz frequency and 5 mT MF as well as alternating MF influence increased sugar beet (*Beta vulgaris* var *saccharifera*) root and leaf yield (Rochalska, 2008); while a dramatic increase in root length, root surface area and root volume was observed in chickpea exposed in batches to static MF of strength from 0 to 250 mT in steps of 50 mT for 1–4 h (Vashisth and Nagarajan, 2008). In the same conditions, seedlings of sunflower showed higher seedling dry weight, root length, root surface area and root volume. Moreover, in germinating seeds, enzyme activities of α-amylase, dehydrogenase and protease were significantly higher in treated seeds than controls (Vashisth and Nagarajan, 2010).

### Effects on gravitropic responses

The growth response that is required to maintain the spatial orientation is called gravitropism and consists of three phases: reception of a gravitational signal, its transduction to a biochemical signal that is transported to the responsive cells and finally the growth response, or bending of root, or shoot. Primary roots exhibit positive gravitropism, i.e., they grow in the direction of a gravitational vector. Shoots respond negatively to gravity and grow upright opposite to the gravitational vector. However, lateral roots and shoots branches are characterized by intermediate set-point angles and grow at a particular angle that can change over time (Firn and Digby, 1997). Gravitropism typically is generated by dense particles that respond to gravity. Experimental stimulation by high-gradient MF provide a new approach to selectively manipulate the gravisensing system.

High-gradient MF has been used to induce intracellular magnetophoresis of amyloplasts and the obtained data indicate that a magnetic force can be used to study the gravisensing and response system of roots (Kuznetsov and Hasenstein, 1996). The data reported strongly support the amyloplast-based gravity-sensing system in higher plants and the usefulness of high MF to substitute gravity in shoots (Kuznetsov and Hasenstein, 1997; Kuznetsov et al., 1999). For example, in shoots of the lazy-2 mutant of tomato that exhibit negative gravitropism in the dark, but respond positively gravitropically in (red) light, induced magnetophoretic curvature showed that lazy-2 mutants perceive the displacement of amyloplasts in a similar manner than wt and that the high MF does not affect the graviresponse mechanism (Hasenstein and Kuznetsov, 1999). Arabidopsis stems positioned in a high MF on a rotating clinostat demonstrate that the lack of apical curvature after basal amyloplast displacement indicates that gravity perception in the base is not transmitted to the apex (Weise et al., 2000). The movement of corn, wheat, and potato (*Solanum tuberosum*) starch grains in suspension was examined with videomicroscopy during parabolic flights that generated 20–25 s of weightlessness. During weightlessness, a magnetic gradient was generated by inserting a wedge into a uniform, external MF that caused repulsion of starch grains. Magnetic gradients were able to move diamagnetic compounds under weightless or microgravity conditions and serve as directional stimulus during seed germination in low-gravity environments (Hasenstein et al., 2013). The response of transgenic seedlings of Arabidopsis, containing either the CycB1-GUS proliferation marker or the DR5-GUS auxin-mediated growth marker, to diamagnetic levitation in the bore of a superconducting solenoid magnet was evaluated. Diamagnetic levitation led to changes that are very similar to those caused by real- [e.g., on board the International Space Station (ISS)] or mechanically-simulated microgravity [e.g., using a Random Positioning Machine (RPM)]. These changes decoupled meristematic cell proliferation from ribosome biogenesis, and altered auxin polar transport (Manzano et al., 2013). Arabidopsis *in vitro* callus cultures were also exposed to environments with different levels of effective gravity and MF strengths simultaneously. The MF itself produced a low number of proteomic alterations, but the combination of gravitational alteration and MF exposure produced synergistic effects on the proteome of plants (Herranz et al., 2013). However, MF leads to redistribution of the cellular activities and this is why application of the proteomic analysis to the whole organs/plants is not so informative.

### Effects on redox status

Effects of MFs have been related to uncoupling of free radical processes in membranes and enhanced ROS generation. It has been experimentally proven that MF can change activities of some scavenging enzymes such as catalase (CAT), superoxide dismutase (SOD), glutathione reductase (GR), glutathione transferase (GT), peroxidase (POD), ascobtate peroxidase (APX), and polyphenoloxidase (POP). Experiments have been performed on several plant species, including pea, land snail (*Helix aspesa*), radish (*Raphanus sativus*), *Leymus chinensis*, soybean, cucumber (*Cucumis stivus*), broad bean, corn, parsley (*Petroselinum crispum*), and wheat (Xia and Guo, 2000; Regoli et al., 2005; Baby et al., 2011; Polovinkina et al., 2011; Anand et al., 2012; Bhardwaj et al., 2012; Jouni et al., 2012; Radhakrishnan and Kumari, 2012, 2013; Shine and Guruprasad, 2012; Shine et al., 2012; Payez et al., 2013; Rajabbeigi et al., 2013; Serdyukov and Novitskii, 2013; Aleman et al., 2014; Haghighat et al., 2014). The results suggest that exposure to increased MF causes accumulation of reactive oxygen species and alteration of enzyme activities. The effects of continuous, low-intensity static MF (7 mT) and EF (20 kV/m) on antioxidant status of shallot (*Allium ascalonicum*) leaves, increased lipid peroxidation and H_2_O_2_ levels in EF applied leaves. These results suggested that apoplastic constituents may work as potentially important redox regulators sensing and signaling MF changes. Static continuous MF and EF at low intensities have distinct impacts on the antioxidant system in plant leaves, and weak MF is involved in antioxidant-mediated reactions in the apoplast, resulting in overcoming a possible redox imbalance (Cakmak et al., 2012). In mung bean seedlings treated with 600 mT MF followed by cadmium stress the concentration of malondialdehyde, H_2_O_2_ and O^−^_2_, and the conductivity of electrolyte leakage decreased, while the NO concentration and NOS activity increased compared to cadmium stress alone, showing that MF compensates for the toxicological effects of cadmium exposure are related to NO signal (Chen et al., 2011).

### Effects on photosynthesis

Photosynthesis, stomatal conductance and chlorophyll content increased in corn plants exposed to static MFs of 100 and 200 mT, compared to control under irrigated and mild stress condition (Anand et al., 2012). Pre-seed electromagnetic treatments has been used to minimize the drought-induced adverse effects on different crop plants. Pretreatment of seeds of two corn cultivars with different magnetic treatments significantly alleviated the drought-induced adverse effects on growth by improving chlorophyll a and photochemical quenching and non-photochemical quenching. Of all magnetic treatments, 100 and 150 mT for 10 min were most effective in alleviating the drought-induced adverse effects (Javed et al., 2011). Polyphasic chlorophyll *a* fluorescence transients from magnetically treated soybean plants gave a higher fluorescence yield. The total soluble proteins of leaves showed increased intensities of the bands corresponding to a larger subunit (53 KDa) and smaller subunit (14 KDa) of Rubisco in the treated plants. Therefore, pre-sowing magnetic treatment was found to improve biomass accumulation in soybean (Shine et al., 2011). Other general effects on MF application on chlorophyll content have been documented for several plant species (Voznyak et al., 1980; Rochalska, 2005; Turker et al., 2007; Radhakrishnan and Kumari, 2013).

The CO_2_ uptake rate of MF exposed radish seedlings was lower than that of the control seedlings. The dry weight and the cotyledon area of MF exposed seedlings were also significantly lower than those of the control seedlings (Yano et al., 2004). A MF of around 4 mT had beneficial effects, regardless of the direction of MF, on the growth promotion and enhancement of CO_2_ uptake of potato plantlets *in vitro*. However, the direction of MF at the MF tested had no effects on the growth and CO_2_ exchange rate (Iimoto et al., 1998).

A permanent MF induces significant changes in bean leaf fluorescence spectra and temperature. The fluorescence intensity ratio (FIR) and change of leaf temperature ΔT increase with the increase of MF intensity. The increase of ΔT due to MFs is explained in bean with a simple ion velocity model. Reasonable agreement between calculated ΔT, based on the model, and measured ΔT was obtained (Jovanic and Sarvan, 2004).

### Effects on lipid composition

In radish seedlings grown in lowlight and darkness in an ELF MF characterized by 50 Hz frequency and approximate to 500 μT flux density, MF exposure increased the production of polar lipids by threefold specifically, glycolipids content increased fourfold and phospholipids content rose 2.5 times, compared to seeds. MF stimulated lipid synthesis in chloroplast, mitochondrial, and other cell membranes (Novitskii et al., 2014). Furthermore, among fatty acids, MF exerted the strongest effect on the content of erucic acid: it increased in the light and in darkness approximately by 25% and decreased in the light by 13%. Therefore, MF behaved as a correction factor affecting lipid metabolism on the background of light and temperature action (Novitskaya et al., 2010).

Plasma membranes of seeds of tomato plants were purified, extracted, and applied to a silicon substrate in a buffer suspension and their molecular structure was studied using X-ray diffraction. While MFs had no observable effect on protein structure, enhanced lipid order was observed, leading to an increase in the gel components and a decrease in the fluid component of the lipids (Poinapen et al., 2013b).

### Other effects

Inflorescences from *Tradescantia* clones subjected to high MF showed pink mutations in stamen hair cells (Baum and Nauman, 1984). Pollen grains of papaya (*Carica papaya*) exposed to MF germinated faster and produced longer pollen tubes than the controls (Alexander and Ganeshan, 1990). In kiwifruit (*Actinidia deliciosa*) MF treatment partially removed the inhibitory effect caused by the lack of Ca^2+^ in the pollen culture medium, inducing a release of internal Ca^2+^ stored in the secretory vesicles of pollen plasma membrane (Betti et al., 2011). Short day strawberry (*Fragaria vesca*) plants treated with MF strengths of 0.096, 0.192, and 0.384 Tesla (T) in heated greenhouse conditions showed increased fruit yield per plant (208.50 and 246.07 g, respectively) and fruit number per plant (25.9 and 27.6, respectively), but higher MF strengths than 0.096 T reduced fruit yield and fruit number. Increasing MF strength from control to 0.384 T also increased contents of N, K, Ca, Mg, Cu, Fe, Mn, Na, and Zn, but reduced P and S content (Esitken and Turan, 2004). The effects of pre-sowing magnetic treatments on growth and yield of tomato increased significantly (*P* < 0.05) the mean fruit weight, the fruit yield per plant, the fruit yield per area, and the equatorial diameter of fruits in comparison with the controls. Total dry matter was also significantly higher for plants from magnetically treated seeds than controls (De Souza et al., 2006).

In the presence of a static MF, the rhythmic leaflet movements of the plant *Desmodium gyrans* tended to slowdown. Leaflets moving up and down in a MF of approximately 50 mT flux density increased the period by about 10% due to a slower motion in the “up” position. Since during this position a rapid change of the extracellular potentials of the pulvinus occurs, it was proposed that the effects could be mediated via the electric processes in the pulvinus tissue (Sharma et al., 2000). Electric process imply ion flux variations. The influence of a high-gradient MF on spatial distribution of ion fluxes along the roots, cytoplasmic streaming, and the processes of plant cell growth connected with intracellular mass and charge transfer was demonstrated (Kondrachuk and Belyavskaya, 2001).

In tomato, a significant delay in the appearance of first symptoms of geminivirus and early blight and a reduced infection rate of early blight were observed in the plants from exposed seeds to increased MFs (De Souza et al., 2006).

Single suspension-cultured plant cells of the Madagascar rosy periwinkle (*Catharanthus roseus*) and their protoplasts were anchored to a glass plate and exposed to a MF of 302 ± 8 mT for several hours. Analysis suggested that exposure to the MF roughly tripled the Young's modulus of the newly synthesized cell wall without any lag (Haneda et al., 2006). *In vitro* tissue cultures of *Paulownia tomentosa* and *Paulownia fortunei* exposed to a magnetic flow density of 2.9–4.8 mT and 1 m s^−1^ flow rate for a period of 0, 2.2, 6.6, and 19.8 s showed increased regeneration capability of *Paulownia* cultures and a shortening of the regeneration time. When the cultures were exposed to a MF with strength of 2.9–4.8 mT for 19.8 s, the regenerated *P. tomentosa* and *P. fortunei* plants dominated the control plants (Yaycili and Alikamanoglu, 2005).

Increase in MF conditions may also affect secondary plant metabolism. The growth of suspension cultures of *Taxus chinensis* var. *mairei* and Taxol production were promoted both by a sinusoidal alternating current MF (50 Hz, 3.5 mT) and by a direct current MF (3.5 mT). Taxol production increased rapidly from the 4th day with the direct current MF but most slowly with the alternating current MF. The maximal yield of Taxol was 490 μg 1^−1^ with the direct current MF and 425 μg 1^−1^ with the alternating current MF after 8 d of culture, which were, respectively, 1.4-fold and 1.2-fold of that without exposure to a MF (Shang et al., 2004).

The biological impact of MF strengths up to 30 Tesla on transgenic Arabidopsis plants engineered with a stress response gene consisting of the alcohol dehydrogenase (*Adh*) gene promoter driving the β-glucuronidase (GUS) gene reporter. Field strengths in excess of about 15 Tesla induce expression of the Adh/GUS transgene in the roots and leaves. From the microarray analyses that surveyed 8000 genes, 114 genes were differentially expressed to a degree greater than 2.5 fold over the control. The data suggest that MF in excess of 15 Tesla have far-reaching effect on the genome. The wide-spread induction of stress-related genes and transcription factors, and a depression of genes associated with cell wall metabolism, are prominent examples. The roles of MF orientation of macromolecules and magnetophoretic effects are possible factors that contribute to the mounting of this response (Paul et al., 2006).

Table 1 summarizes the effects of low and high intensity MF on plants.

## The geomagnetic field and plant evolution

**Figure 1:**
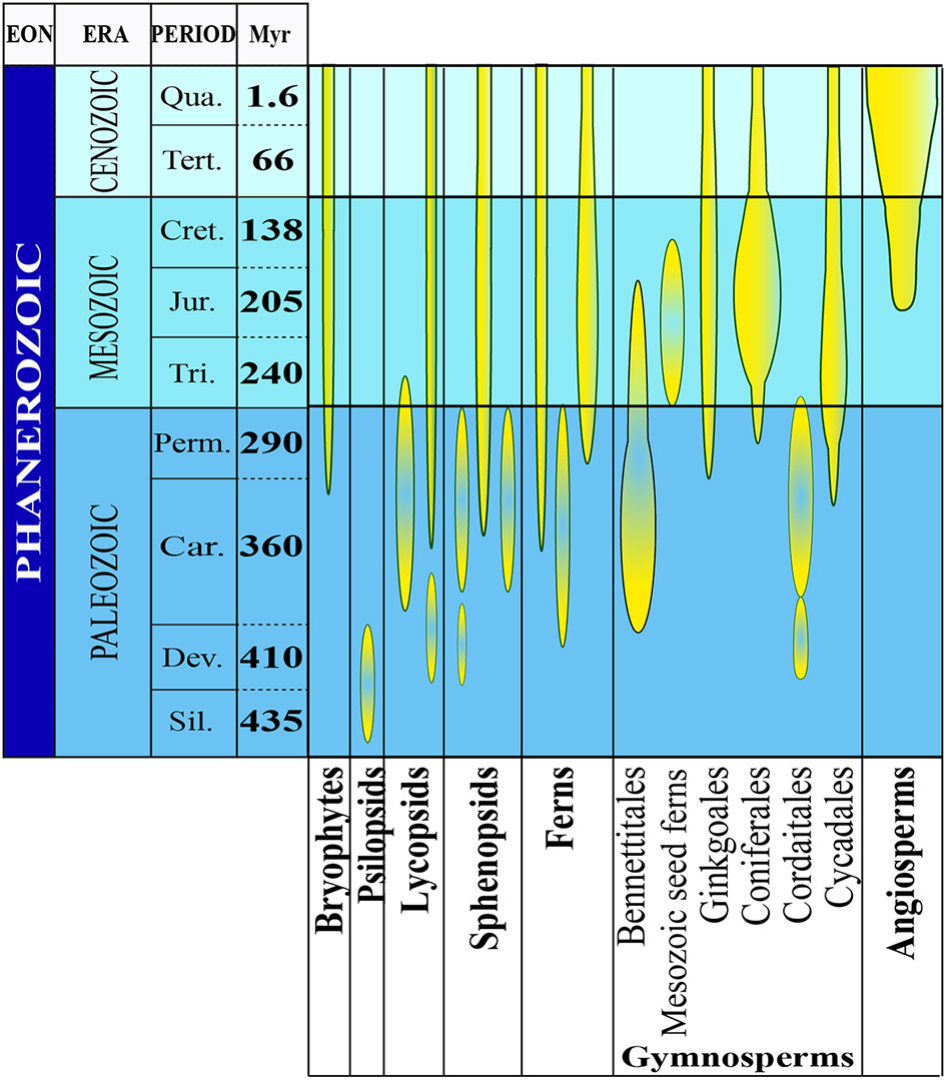
**The evolutionary history of plants**. The abundance and diversity of plant fossils increase in the Silurian Period where the first macroscopic evidence for land plants has been found. There is evidence for the evolution of several plant groups of the late Devonian and early Carboniferous periods (homosporous ferns and gymnosperms). From the late Devonian through the base of the late Cretaceous period, gymnosperms underwent dramatic evolutionary radiations and became the dominant group of vascular plants in most habitats. Flowering plants probably also originated during this time, but they did not become a significant part of the fossil flora until the middle of the Cretaceous Period (Modified from Occhipinti et al., 2014).

Along with gravity, light, temperature and water availability, the GMF has been present since the beginning of plant evolution. Apart from gravity, all other factors, including the GMF, changed consistently during plant evolution thereby representing important abiotic stress factors eventually contributing to plant diversification and speciation. Mass-extinction events profoundly reshaped Earth's biota during the early and late Mesozoic and terrestrial plants were among the most severely affected groups. Several plant families were wiped out, while some new families emerged and eventually became dominant (Figure 1).

**Figure 2:**
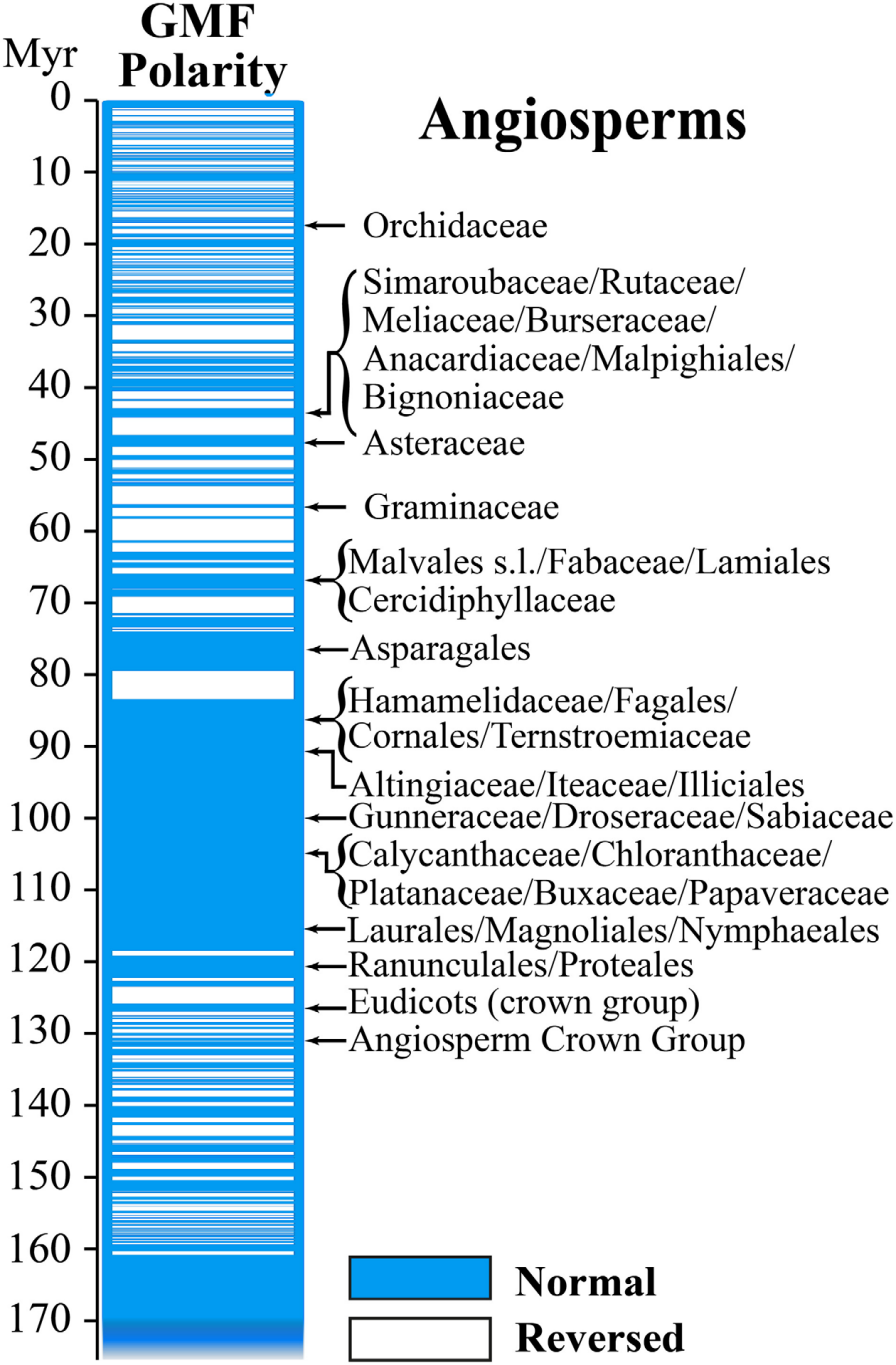
**Geomagnetic field reversals and Angiosperm evolution**. In the direct comparison of GMF polarity and diversion of Angiosperms it is interesting to note that most of the diversion occurred during periods of normal magnetic polarity (Modified from Occhipinti et al., 2014).

The behavior of the GMF during the Mesozoic and Late Paleozoic, or more precisely between 86 and 276.5 millions of years (Myr), is of particular interest. Its virtual dipole moment (VDM) seems to have been significantly reduced (≈4 × 10^22^ Am^2^) compared to today's values (Shcherbakov et al., 2002). In Earth's history, the GMF exhibited several changes of magnetic polarity, with the so-called geomagnetic reversals or excursions, characterized by persistent times with the same polarity. They occurred some hundred times since Earth formation and the mean time between a reversal and the next one has been estimated around 300,000 years (Figure 2). Because the present normal polarity started around 780,000 years ago and a significant field decay has been occurring during the last 1000 years, an imminent geomagnetic reversal would not be so unexpected (De Santis et al., 2004).

In the course of biological evolution, the fossil record tells us mass extinction occurred several times. One of working hypothesis to explain such mass extinctions is the cease of the GMF when the geomagnetic pole was reversed. Because the strength of the GMF is strongly reduced during polarity transitions, when compared to stable normal or reversed polarities, we recently propose that these variations might be correlated to plant evolution (Occhipinti et al., 2014). We do not have measurable records of GMF polarity reversal before late Jurassic, therefore we can only compare variations of GMF polarity with diversion of families and orders of Angiosperms in the Tertiary and Cretaceous periods. Angiosperms are regarded as one of the greatest terrestrial radiations of recent geological times. The oldest Angiosperm fossils date from the early Cretaceous, 130–136 Myr ago, followed by a rise to ecological dominance in many habitats before the end of the Cretaceous (Soltis et al., 2008). It has been shown that the periods of normal polarity transitions overlapped with the diversion of most of the familial angiosperm lineages (Figure 2). This correlation appears to be particularly relevant to Angiosperms compared to other plants (Occhipinti et al., 2014).

Patterns of diversification reconstructed onto phylogenetic trees depend on the age of lineages, their intrinsic attributes, and the environments experienced since their origins. Global environments have changed considerably during the history of angiosperm radiation; e.g., the rise of grasses to dominance during the late Tertiary has been linked to global cooling and drying. The greater incidence of high-energy particles, and direct effects of magnetism on biological system during reversals period might contribute to alteration that eventually led to mass extinction. Because plants, in general, do not change their orientation once germinated, there might be distinctive action of the terrestrial magnetism on the growth and physiology of plants (Yamashita et al., 2004). Magnetoreception might be a driving force contributing to plant evolution, but in order to prove such hypothesis we should be able to demonstrate that some plant genes are affected by MF reversals.

## Possible mechanisms of magnetoreception

For a number of years laboratory studies on the biological effects of MF have demonstrated that the fields can produce or alter a wide range of phenomena. Explaining the diversity of the reported effects is a central problem. In recent years, the following types of physical processes or models underlying hypothetically primary mechanisms of the interaction of MF responses in biological systems have been proposed: (a) classical and quantum oscillator models; (b) cyclotron resonance model; (c) interference of quantum states of bound ions and electrons; (d) coherent quantum excitations; (e) biological effects of torsion fields accompanying MF; (f) biologically active metastable states of liquid water; (g) free-radical reactions and other “spin” mechanisms; (h) parametric resonance model; (i) stochastic resonance as an amplifier mechanism in magnetobiology and other random processes; (j) phase transitions in biophysical systems displaying liquid crystal ordering; (k) bifurcation behavior of solutions of non-linear chemical kinetics equations; (l) radio-technical models, in which biological structures and tissues are portrayed as equivalent electric circuits and; (m) macroscopic charged vortices in cytoplasm. Furthermore, mechanisms combining these concepts and models cannot be excuded (Belyavskaya, 2004).

Observation of resonance effects at specific frequencies, combined with new theoretical considerations and calculations, indicate that birds use a radical pair with special properties that is optimally designed as a receptor in a biological compass. This radical pair design might be realized by cryptochrome photoreceptors if paired with molecular oxygen as a reaction partner (Ritz et al., 2009, 2010). Therefore, several considerations have suggested that cryptochromes are likely to be the primary sensory molecules of the light-dependent magnetodetection mechanism, which has been suggested to be radical pair based (Liedvogel and Mouritsen, 2010).

In plants, cryptochromes control different aspects of growth and development, i.e., involvement in de-etiolation responses such as inhibition of hypocotyl growth (Ahmad and Cashmore, 1993; Lin, 2002), anthocyanin accumulation (Ahmad et al., 1995) leaf and cotyledon expansion (Cashmore et al., 1999; Lin, 2002), transitions to flowering (El-Assal et al., 2003) or regulation of blue-light regulated genes (Jiao et al., 2003). In Arabidopsis, cryptochromes are encoded by two similar genes, *cry1* and *cry2*. CRY2 protein levels in seedlings decrease rapidly upon illumination by blue light, presumably as a result of protein degradation of the light-activated form of the receptor (Ahmad et al., 2007). Like photolyases, plant cryptochromes undergo a light-dependent electron transfer reaction, known as photoactivation, that leads to photoreduction of the flavin cofactor, FAD (Giovani et al., 2003).

Particular attention has been paid to the potential role of cryptochrome as a plant magnetosensor (Ahmad and Cashmore, 1993; Ang et al., 1998; Chattopadhyay et al., 1998; Mockler et al., 1999; Lin, 2002; El-Assal et al., 2003; Giovani et al., 2003; Jiao et al., 2003; Zeugner et al., 2005; Ahmad et al., 2007; Bouly et al., 2007; Kleine et al., 2007; Solov'yov et al., 2008; Harris et al., 2009; Liedvogel and Mouritsen, 2010; Ritz et al., 2010; Solov'yov and Schulten, 2012).

Experiments on Arabidopsis have suggested that magnetic intensity affects cryptochrome-dependent growth responses (Ahmad et al., 2007). But, as discussed above, these reported cryptochrome-mediated MF effects on plant growth could not be replicated in an independent study (Harris et al., 2009). These findings would be very important, if they turn out to exist and be independently replicable, since even though magnetic responses do not seem biologically relevant for the plant, they would show in principle that biological tissue is sensitive to the MF responses that are linked to cryptochrome-dependent signaling pathways. They could thus confirm the ability of cryptochrome to mediate MF responses (Liedvogel and Mouritsen, 2010).

The claimed magnetosensitive responses can best be explained by the radical pair model, as Arabidopsis cryptochromes form radical pairs after photoexcitation (Giovani et al., 2003; Zeugner et al., 2005; Bouly et al., 2007) and these experiments might reflect common physical properties of photoexcited cryptochromes in both plants and animals.

The radical-pair mechanism is currently the only physically plausible mechanism by which magnetic interactions that are orders of magnitude weaker than the average thermal energy, *k*_B_T, can affect chemical reactions. The kinetics and quantum yields of photo-induced flavin-tryptophan radical pairs in cryptochrome are indeed magnetically sensitive and cryptochrome is a good candidate as a chemical magnetoreceptor. Cryptochromes have also attracted attention as potential mediators of biological effects of ELF EMFs and possess properties required to respond to Earth-strength (approximately 50 μT) fields at physiological temperatures (Maeda et al., 2012).

**Figure 3:**
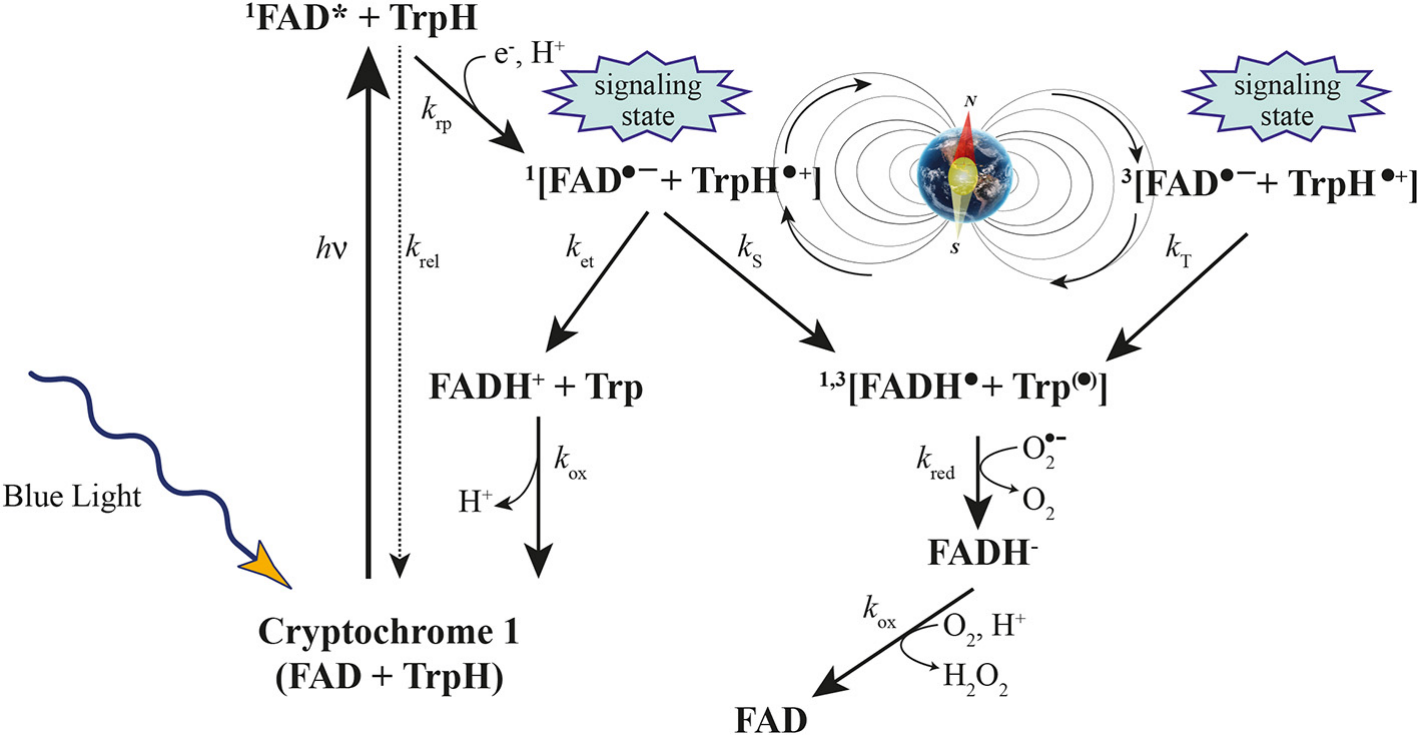
**Cryptochrome activation and inactivation reactions**. Blue light activates cryptochrome through absorbing a photon by the flavin cofactor. FAD becomes promoted to an excited FAD^*^ state and receives an electron from a nearby tryptophan, leading to the formation of the [FADH• + Trp•] radical pair, which exists in singlet ^(1)^ and triplet ^(3)^ overall electron spin states by coherent geomagnetic field-dependent interconversions. Under aerobic conditions, FADH• slowly reverts back to the initial inactive FAD state through the also inactive FADH^−^ state of the flavin cofactor (Modified from Occhipinti et al., 2014).

Recently, a combination of quantum biology and molecular dynamics simulations on plant cryptochrome has demonstrated that after photoexcitation a radical pair forms, becomes stabilized through proton transfer, and decays back to the protein's resting state on time scales allowing the protein, in principle, to act as a radical pair-based magnetic sensor (Solov'yov and Schulten, 2012 and references therein) (Figure 3). Furthermore, the elimination of the local GMF weakens the inhibition of Arabidopsis hypocotyl growth by white light, and delays flowering time. The expression changes of three Arabidopsis cryptochrome-signaling-related genes, (PHYB, CO, and FT) suggest that the effects of a near-null MF are cryptochrome-related, which may be revealed by a modification of the active state of cryptochrome and the subsequent signaling cascade plant cryptochrome has been suggested to act as a magnetoreceptor (Xu et al., 2012).

## Conclusion and perspectives

Revealing the relationships between MF and plant responses is becoming more and more important as new evidence reveals the ability of plants to perceive and respond quickly to varying MF by altering their gene expression and phenotype. The recent implications of MF reversal with plant evolution opens new horizons not only in plant science but also to the whole biosphere, from the simplest organisms to human beings.

Magnetotactic bacteria are a diverse group of microorganisms with the ability to orient and migrate along GMF lines (Yan et al., 2012); the avian magnetic compass has been well-characterized in behavioral tests (Ritz et al., 2009); magnetic alignment, which constitutes the simplest directional response to the GMF, has been demonstrated in diverse animals including insects, amphibians, fish, and mammals (Begall et al., 2013); concerns of possible biological effects of environmental EMFs on the basis of the energy required to rotate the small crystals of biogenic magnetite that have been discovered in various human tissues have been discussed (Kobayashi and Kirschvink, 1995). The overall picture is thus a general effect of GMF on life forms.

Life evolved on Earth along changes in the GMF life-history. Any other environment lacking a GMF is expected to generate reactions in living organisms. These concerns becomes urgent questions in light of planned long-term flights to other planets (Belyavskaya, 2004). Understanding GMF effects on life will provide the fundamental background necessary to understand evolution of life forms in our planet and will help us to develop scientific recommendations for design of life-support systems and their biotic components for future space exploration.

### Conflict of interest statement

The author declares that the research was conducted in the absence of any commercial or financial relationships that could be construed as a potential conflict of interest.
